# HIV—Now Another Long Term Chronic Condition?

**DOI:** 10.3390/v18030283

**Published:** 2026-02-27

**Authors:** Julian W. Tang, Dariusz P. Olszyna, Iain Stephenson, Sophia Archuleta

**Affiliations:** 1 Clinical Microbiology, University Hospitals of Leicester NHS Trust, Leicester LE1 5WW, UK; 2 Division of Infectious Diseases, Department of Medicine, National University Hospital, Singapore 119074, Singapore; 3 Department of Medicine, Yong Loo Lin School of Medicine, National University of Singapore, Singapore 119228, Singapore; 4 Infectious Diseases Unit, University Hospitals of Leicester NHS Trust, Leicester LE1 5WW, UK

**Keywords:** HIV, PLWH, chronic disease, treatment, adherence, cardiovascular, renal, respiratory, neurology, ageing

## Abstract

Over the past 25 years, with advances in treatment, HIV infection has become a manageable, chronic illness. In patients who manage their treatment carefully, they can expect almost normal lifespans, similar to other chronic diseases like diabetes. This article briefly summarizes ongoing risks to people living with HIV, highlighting the need to adhere to therapy and monitoring for possible long-term complications and adverse effects in the context of other common systemic illnesses.

Over the past 25 years, advances in antiretroviral therapy (ART) have revolutionized the management of HIV infection, transforming it from a terminal diagnosis into a chronic illness comparable to hypertension or diabetes. People living with HIV (PLWH) now enjoy a relatively normal quality of life and lifespan, provided that adherence with ART is maintained, along with routine monitoring of viral load and CD4 counts. The most recent development—the use of long-acting, injectable ARTs [1]—has made HIV infection even more similar to other chronic conditions, such as diabetes, rather than the death sentence that it was in the late 1980s and early 1990s.

Other parallels with diabetes can be made, where the underlying process as well as its treatment can cause long-term complications if the balance is not optimal for individual patients. Poor glycemic control in diabetes can lead to multi-system complications (e.g., cardiomyopathy, nephropathy, retinopathy, peripheral neuropathy) [2]. Poor virologic control in HIV infection can lead to reduction in CD4 T-cells and host immunity resulting in opportunistic infections and reduced quality of life (e.g., *Candida*, *Pneumocystis*, cytomegalovirus and JC virus infections) [3]. Poorly adjusted oral or injected anti-diabetic (e.g., oral hypoglycemic drugs and insulin) can cause hypo- or hyper-glycemic crises. Suboptimal HIV treatment regimens can lead to drug resistance and viral rebound. This can occur from poor adherence due to unexpected adverse effects, unforeseen or unplanned treatment in interruptions (e.g., during vacations) or pharmacokinetic drug interactions leading to subtherapeutic drug levels.

Advances in HIV management mean that there are increasingly more people living, and also ageing, with HIV. This presents opportunities for promoting healthy longevity in this unique population, and additional challenges for health systems that have previously been used to responding to acute opportunistic infections and other AIDS-related complications. Individual HIV practitioners may also need to re-think their services and integrate screening for age-related issues, such as frailty and cognitive decline, into the primary care of people living with HIV.

## 1. HIV—The Pathogenesis of Living Longer

Modern HIV drugs, when given early in the infection suppress viral replication and limit the damage to the host immune system, allowing it to function more normally. This maintains CD4/CD8 lymphocyte counts and reduces the risk of opportunistic infections. A key aim is early identification and treatment of HIV infection. Late diagnosis of long-term infections generally results in poorer outcomes and lower life expectancies. Improved outcomes also depend on adherence with the treatment regimen, which is supported by patient engagement and community support. As the virus still cannot be eradicated, only strict control of its replication will lead to longer, healthier lifespans. Another benefit of sustained virological control following adherence to treatment is the reduction in viral loads to undetectable levels that are untransmissible by sexual activity with partners. Thus, antiviral therapy is both treatment and prevention.

Given the above, the long-term survival for those who are treated early and remain engaged with HIV care depends on how their care interacts with their lifestyles and any other pre-existing comorbidities, e.g., obesity, diabetes, cardiovascular diseases (ischemic heart disease—IHD, hypertension, stroke), respiratory diseases (asthma, chronic obstructive pulmonary disease—COPD), neurological diseases (dementia, Parkinson’s disease), chronic renal and liver failure, etc. [4,5,6].

Socioeconomic factors also become more relevant with longer life expectancies [7]. Whilst healthcare in the UK is free at point of care (though drug prescription charges need to be paid by most patients) under the National Health Service (NHS), this is not necessarily the case elsewhere. Costs can accumulate with HIV-related clinic visits, in addition to those for other comorbidities. It is known that chronic socioeconomic pressures can shorten the lives of those on lower incomes.

## 2. Risk Factors for Cardiovascular Disease in PLWH

HIV and potentially some antiretrovirals such as abacavir, can increase inflammation in vessel walls and damage the endothelium. Even with successful viral suppression, HIV infection itself, as well as the use of protease inhibitors can promote dyslipidemia, which also increases the risk of atherosclerosis and elevates long-term cardiovascular risk including IHD, hypertension and stroke. Cardiovascular disease risk may also be compounded by smoking and drug and alcohol use. Injection drug use risks coinfection with other bloodborne viruses, like hepatitis B and C [4,6].

## 3. Risk Factors for Respiratory Diseases in PLWH

PLWH may also be exposed to respiratory infections like tuberculosis (TB), and opportunistic infections like *Pneumocystis jirovecii pneumonia* (PJP) and cytomegalovirus (CMV) although antiretroviral treatment with immune restoration to more normal CD4 counts reduces their risk. COPD is a risk for smokers, regardless of HIV status, so smoking cessation should be encouraged. HIV-related ongoing inflammation may affect the response to other seasonal infections like viral and bacterial causes of community-acquired pneumonia (CAP). PLWH should receive COVID, pneumococcal and seasonal influenza vaccination (as a high-risk group), along with early detection and treatment of opportunistic infections [4,6].

## 4. Risk Factors for Neurological Diseases in PLWH

Ongoing neuroinflammation is also part of the HIV infection process. HIV proteins, TAT, VPR and gp120 may contribute directly to this via oxidative stress and excitotoxicity (excessive glutamate, N-methyl-D-aspartate (NMDA) release and signalling). Some antiretroviral drugs can also have direct neurological adverse effects that can contribute to this pathology over time [7].

Reduced immune control may allow more frequent and prolonged reactivation of neurotropic herpes viruses like herpes simplex viruses 1 and 2 (HSV-1/2) and varicella zoster virus (VZV), which have been associated with earlier diagnoses of Alzheimer’s dementia. HIV-associated neurocognitive decline (HAND) is also well-recognized in PLWH, and can be reduced with viral suppression, though not eliminated completely. HIV-related damage to the blood–brain barrier and ongoing neuroinflammation can still increase the progression of PLWH towards dementia, despite effective viral suppression [8]. Other neurological conditions like Parkinson’s, may also be accelerated due to these neuroinflammatory processes in the basal ganglia, damaging the dopaminergic centres [5].

## 5. Risk Factors for Other Diseases of Ageing in PLWH

Chronic renal and liver disease, arthritis, and cancer risks remain slightly elevated in PLWH. This may be a founder effect where an earlier, unhealthy lifestyle has laid the basis for some of these later chronic conditions. However, HIV infection and ARTs may also have cumulative effects that increase these lifetime risks above those of the general population.

Drug elimination of ARTs via the renal or hepatic route may lead to toxicity over many years. HIV-associated nephropathy (HIVAN) is well-recognized, as well as immune-complex deposition, thrombotic microangiopathy, which also add to renal dysfunction, and may all be linked to chronic HIV-related systemic inflammation [4,6]. Age, pre-existing comorbidities (like obesity, hypertension, diabetes), lifestyle factors (smoking, recreational drug and alcohol use) also contribute.

Hepatitis B (HBV) and/or hepatitis C (HCV) coinfections are more common in PLWH, where untreated coinfections can lead to hepatic fibrosis, cirrhosis and hepatocellular carcinoma [4,6]. In addition, there is increasing evidence that HCV can cause many varied neurological conditions; therefore, detection, control and ultimately elimination of coinfecting HBV and/or HCV should be a priority.

Reduced immune control of latent oncogenic viruses (HBV, human papilloma virus —HPV, Epstein–Barr virus—EBV, Kaposi’s sarcoma virus/human herpesvirus 8—KSHV/HHV8) that are more prevalent in PLWH due to overlapping transmission risk, can lead to increased cancer risks in PLWH—even in those with effective viral suppression. Ongoing systemic inflammation (i.e., the damage–repair cycle) and residual host immune damage from HIV can also increase the overall background risk of cancer, e.g., by reducing the effectiveness of cancer surveillance, as part of the ongoing immune dysregulation. HIV itself might have a direct pro-oncogenic effect, and some ART drugs may increase the lifetime cancer risk [9].

The normal ageing and lifestyle-related (alcoholism, poor nutrition, long-term recreational drug-use) damage to joints and the musculoskeletal system in PLWH is further exacerbated by the background of ongoing systemic inflammation and adverse ART drug effects. In addition, metabolic changes related to HIV and ARTs, together with immune dysregulation and dyslipidemia, can contribute to osteoporosis and osteonecrosis, as well as unmasking some underlying rheumatological conditions. Episodes of immune reconstitution inflammatory syndrome (IRIS) and recovery from them can also damage muscle and joints and reveal other undiagnosed autoimmune conditions [10].

## 6. Conclusions

PLWH are living longer, healthier lives thanks to ever-evolving, more effective ART. Yet these gains rely on continued engagement and adherence with HIV treatment, healthy lifestyle promotion, and the close monitoring for, and the prompt management of, other non-HIV-related disease conditions, including any ART adverse drug effects.

## Data Availability

Not applicable.
